# Hypoxia-Inducible Factor-1α Stabilization Alleviates Hypoxia-Induced Temporomandibular Joint Osteoarthritis by Activating MDM2 to Suppression of p53 Signalling

**DOI:** 10.1016/j.identj.2026.109805

**Published:** 2026-08-22

**Authors:** Zhonghan Chen, Jingwen Cai, Jiayi Cai, Xinhan Yang, Zhenling Wang, Linxin Chen, Sihang Chen, Linyu Xu, Han Wang

**Affiliations:** a Clinical Research Center for Oral Tissue Deficiency Diseases of Fujian Province & Fujian Key Laboratory of Oral Diseases & Fujian Provincial Engineering Research Center of Oral Biomaterial, School and Hospital of Stomatology, Fujian Medical University, Fuzhou, Fujian, China; b Stomatological Key Laboratory of Fujian College and University & Institute of Stomatology & Department of Orthodontics, School and Hospital of Stomatology, Fujian Medical University, Fuzhou, Fujian, China

**Keywords:** Temporomandibular joint disorders, Temporomandibular joint, Hypoxia, Hypoxia-inducible factor-1α

## Abstract

**Introduction and aims:**

Temporomandibular joint osteoarthritis (TMJOA) is characterized by cartilage degradation and chronic inflammation. Although hypoxia is recognized to drive the pathological process in osteoarthritis, its underlying mechanisms in TMJOA remain elusive. This study aimed to elucidate the roles of hypoxia and hypoxia-inducible factor-1α (HIF-1α) in TMJOA.

**Methods:**

A rat TMJOA model was established, and a hypoxia probe confirmed a hypoxic niche in condylar cartilage. Primary chondrocytes were exposed to hypoxia, with HIF-1α stabilized by dimethyloxaloylglycine (DMOG) and validated using the p53 agonist NSC-207895. Transcriptomic profiling identified downstream pathways. Coimmunoprecipitation, chromatin immunoprecipitation-qPCR, and proteasome inhibitor MG132 intervention further dissected the ubiquitin-dependent regulatory mechanism between HIF-1α, MDM2, and p53.

**Results:**

A hypoxic niche was detected in early TMJOA prior to overt degeneration. Hypoxia strongly induced inflammatory responses and matrix degradation in chondrocytes. Stabilization of HIF-1α by DMOG rescued these catabolic effects in vitro and significantly alleviated TMJOA pathology in vivo. Transcriptomic, pharmacological, and biochemical assays demonstrated that HIF-1α confers chondroprotection via MDM2-mediated suppression of the p53 signalling pathway: stabilized HIF-1α transcriptionally activates *Mdm2*, promoting p53 ubiquitination and proteasomal degradation to dampen downstream catabolic outputs.

**Conclusions:**

Early TMJOA is accompanied by a pathological hypoxic microenvironment in condylar cartilage. Stabilization of HIF-1α exerts a chondroprotective effect by transcriptionally activating *Mdm2* to suppress p53 signalling, thereby reducing hypoxia-induced inflammation and matrix degradation. The HIF-1α/MDM2/p53 axis may serve as a promising therapeutic target for early-stage TMJOA.

**Clinical relevance:**

TMJOA lacks effective disease-modifying treatments. Our findings highlight the HIF-1α/MDM2/p53 axis as a novel target, with prolyl hydroxylase inhibitors such as DMOG offering potential for noninvasive early intervention to slow disease progression.

## Introduction

Temporomandibular joint osteoarthritis (TMJOA) is a widespread chronic degenerative disorder that severely compromises jaw function, causing debilitating pain and disability.^1^ Pathologically, it is defined by progressive cartilage degradation, subchondral bone remodelling, and synovial inflammation.^2^ Given the increasing global prevalence and associated socioeconomic burden, there is an urgent need for effective therapeutic strategies.^3^ However, current treatments remain largely palliative, as the molecular mechanisms governing TMJOA initiation and early progression particularly the role of the hypoxic microenvironment remain elusive.^4^ Notably, the TMJ presents unique research and translational challenges: its distinct anatomical features (fibrous cartilage, limited vascularity) and high magnitude masticatory biomechanical forces create a hypoxia-dominated microenvironment distinct from appendicular joints.^5^, ^6^, ^7^ While appendicular joint findings advance osteoarthritis research, hypoxia-driven regulatory pathways including hypoxia-inducible factor-1α (HIF-1α) signalling in TMJOA require rigorous validation within the context of TMJ pathology.^8^^,^^9^

Articular cartilage is inherently avascular, and chondrocytes reside in a physiologically hypoxic microenvironment (approximately 1%-5% O₂).^10^^,^^11^ Pathological hypoxia is increasingly recognized as a driver of catabolic and inflammatory responses in osteoarthritis,^12^ with growing evidence implicating HIF-1α as a key mediator of these pathological effects. Contrary to its homeostatic function, HIF-1α has been linked to ferroptosis and disease exacerbation in OA chondrocytes, similar to its homolog EPAS1 (HIF-2α), a known inducer of catabolic mediators like MMP13.^13^ Consistent with this pathogenic potential, inhibiting HIF-1α with capsiate has been shown to reduce ferroptosis and mitigate OA development.^14^, ^15^, ^16^ Nevertheless, extensive research has firmly established HIF-1α as a critical homeostatic regulator within this hypoxic niche, where it sustains chondrocyte survival by promoting glycolytic metabolism to meet energy demands in the avascular environment, maintains extracellular matrix homeostasis by upregulating anabolic genes such as COL2A1 and aggrecan, and protects chondrocytes from oxidative stress and excessive apoptosis.^17^^,^^18^ This protective role, however, appears to be highly context-dependent, creating a well-documented functional duality in the HIF family. Based on the above, the molecular determinants that govern whether the HIF-1α axis acts protectively or pathologically, particularly during the early, potentially reversible stage of TMJOA, remain unresolved.

In articular cartilage, the tumour suppressor transcription factor p53 plays a decisive role in how chondrocytes respond to mechanical, oxidative, and inflammatory stress.^19^^,^^20^ When chondrocytes are exposed to aging, biomechanical overload, or inflammatory mediators, p53 expression is induced, leading to activation of downstream effectors (eg, p53AIP1, PUMA) and triggering apoptosis, senescence, and extracellular matrix degradation.^21^^,^^22^ Additionally, studies have shown that modulation of p53 can alleviate the senescence-associated secretory phenotype in osteoarthritis by regulating the STAG1/Tp53/P21 signalling pathway.^23^ Moreover, recent investigations have demonstrated that downregulating p53 expression in aged chondrocytes leads to a reduction in senescence-associated gene expression and the restoration of matrix homeostasis.^24^ The suppression of p53 may alleviate age-related and inflammation-induced chondrocyte apoptosis, thereby potentially preventing cartilage degradation.^25^ Based on recent studies,^26^, ^27^, ^28^ p53 and HIF-1α exhibit a complex dependent relationship in regulating cellular fate. In various cell types, including fibroblasts, the balance between autophagy and apoptosis depends on the dominant expression of either HIF-1α or p53. When HIF-1α is upregulated, it typically promotes autophagy and cell survival, while p53 activation triggers apoptosis and suppresses autophagy. Interestingly, HIF-1α has been shown to antagonize p53-mediated apoptosis by promoting the degradation of the p53 activator HIPK2.^29^ Emerging evidence from asthma models demonstrates that hypoxia-induced HIF-1α stabilization promotes p53 ubiquitination and proteasomal degrap-regulation of the E3 ubiquitin ligase MDM2, thereby inhibiting apoptosis and maintaining cell viability.^30^ This reciprocal antagonism suggests that HIF-1α activation could function as a natural brake on p53-driven catabolic and apoptotic pathways. Although the HIF-1α–p53 axis has been predominantly studied in tumour biology, its regulatory dynamics in the TMJOA remain underexplored.

Given the controversial role of HIF-1α in TMJOA regulation and its potential crosstalk with the p53 pathway during cellular stress, this study investigated whether stabilizing HIF-1α under early pathological hypoxia modulates p53 signalling to attenuate inflammation. We employed an early-stage unilateral anterior crossbite (UAC) rat model^12^^,^^31^ to rapidly induce characteristic TMJ degeneration, complemented by in vitro hypoxia assays (1% O₂) using primary chondrocytes. We integrated structural, molecular, and biochemical analyses to delineate this regulatory axis. We found that HIF-1α stabilization transcriptionally upregulates *Mdm2*, promoting p53 ubiquitination and destabilization, thereby alleviating hypoxia-induced catabolic and inflammatory responses. These findings establish the HIF-1α/MDM2/p53 axis as a tractable therapeutic target for early-stage TMJOA intervention.

## Materials and methods

### Experimental animals and model establishment

UAC model was established as previously described.^32^ Briefly, rats were anesthetized by intraperitoneal injection of pentobarbital sodium (45 mg/kg), and metal crowns were carefully bonded to the maxillary and mandibular incisors to induce an anterior crossbite relationship when the mouth was closed. Rats in the control group underwent the same anaesthesia and feeding procedures but did not receive crown bonding. The crowns were checked daily to ensure stability, and if dislodged, they were immediately rebonded.

### Animals and experimental design

Thirty 5-week-old male Sprague–Dawley (SD) rats were exclusively selected to eliminate confounding interference from cyclic female sex hormones that disrupt HIF signalling and TMJ chondrocyte homeostasis.^33^ All procedures were approved by the Animal Care and Use Committee of Fujian Medical University (IACUC FJMU 2025-Y-0176). The rats were housed under a 12-hour light/dark cycle at room temperature with a relative humidity of 55% ± 5% and were acclimatized for 1 week prior to the experiments. The study was conducted in two independent batches, each with a 2-week intervention period. In the first batch, rats were randomly assigned to either the control group (*n* = 6) or the UAC group (*n* = 6). In the second batch, rats were randomly allocated into three groups: sham (*n* = 6), UAC (*n* = 6), and UAC + dimethyloxaloylglycine (DMOG) (*n* = 6). Normal saline was injected into the articular cavity in the sham group and the UAC group. In the UAC + DMOG group, intra-articular injections of DMOG (1 mM) were administered into the temporomandibular joint every other day for 2 weeks, with 50 μL injected each time. At the end of each intervention, rats were sacrificed for sample collection.

### Micro-CT scanning evaluation

TMJ tissues were excised and fixed in 4% paraformaldehyde for 24 hours. The specimens were subsequently scanned using a high-resolution NEMO Micro-CT system (model NMC-200), manufactured by Pingseng Medical Technology. Scanning parameters were set at 70 kV voltage, 200 μA current, and high resolution. Images were processed with Cruiser software and reconstructed with the Avatar system to generate 3D trabecular bone models. Three cubic regions of interest (each 0.5 × 0.5 × 0.5 mm³) were selected 0.5 mm below the condylar subchondral bone. In these regions, parameters were calculated: bone volume fraction (BV/TV), trabecular thickness (Tb.Th), trabecular number (Tb.N), and trabecular separation (Tb.Sp).

### Histological observation

The specimens were decalcified in 10% ethylenediaminetetraacetic acid (EDTA) for approximately 2 months, dehydrated, embedded in paraffin, and sagittal sectioning at a thickness of 3 μm. Subsequently, serial sections were subjected to haematoxylin and eosin (H&E), Safranin O/fast green (S&F), and toluidine blue staining to evaluate morphological changes, cartilage integrity, proteoglycan distribution, and protein expression, respectively.

### Immunohistochemistry

Following deparaffinization and antigen retrieval, tissue sections were incubated overnight at 4°C with primary antibodies against COL2A1 (1:1000; Servicebio), MMP13 (1:500; Abcam), HIF-1α (1:50; Novusbio), and p53 (1:200; ABclonal). After washing, sections were incubated with a biotinylated goat antirabbit secondary antibody (1:500; Boster) for 30 minutes, followed by HRP-conjugated streptavidin. Signals were developed with a diaminobenzidine (DAB) kit and counterstained with haematoxylin. Images were acquired under a light microscope (Nikon Eclipse Ci-L) with a 20× objective. Using ImageJ, the DAB-positive area was quantified after colour deconvolution, and the percentage of positively stained area was calculated as (DAB-positive area/total cartilage area) × 100%.

### Hypoxia detection

Tissue hypoxia was assessed using the Hypoxyprobe-1 Omni Kit (Hypoxyprobe Inc). Following the manufacturer’s instructions, pimonidazole hydrochloride was freshly prepared in sterile saline and administered intraperitoneally at a dose of 60 mg/kg body weight. One hour postinjection, the rats were sacrificed, and the TMJ tissues were harvested. The specimens were fixed in 4% paraformaldehyde, decalcified, embedded in paraffin, and sectioned. Immunohistochemistry was subsequently performed using an affinity-purified rabbit antipimonidazole antibody (1:50 dilution) provided with the kit, followed by a horseradish peroxidase-conjugated secondary antibody and visualization with diaminobenzidine (DAB). Counterstaining was conducted with haematoxylin.

### Isolation and culture of mandibular condylar chondrocytes (MCCs)

Mandibular condylar chondrocytes were isolated from 7-day-old SD rats, adhering to the guidelines of Fujian Medical University. The mandibular condylar cartilage was meticulously dissected and rinsed twice with Dulbecco’s modified Eagle’s medium (DMEM; Gibco) supplemented with 1% penicillin-streptomycin (Hyclone), prior to undergoing sequential enzymatic digestion. Initially, tissues were treated with 0.25% trypsin (Hyclone) at 37°C for 30 minutes, followed by 0.2% collagenase II (Sigma) for 4 hours at the same temperature. The isolated chondrocytes were subsequently cultured in DMEM enriched with 10% foetal bovine serum (Gibco) at 37°C within a humidified incubator supplied with 5% CO₂. Cells from passages 1 to 3 were utilized for subsequent experiments. For DMOG treatment in vitro, gradient concentrations (0, 0.1, 0.5, 1, 2, 4 mM) were screened via CCK-8 cell viability assay (Figure S1B). 1 mM DMOG was selected as the working concentration because it exerted no obvious cytotoxicity on MCCs and efficiently induced stable HIF-1α accumulation.

### Hypoxic cell model

Cells in the logarithmic growth phase were harvested by digestion with 0.25% trypsin and centrifuged. The cell pellet was resuspended in fresh culture medium and seeded into culture plates. To ensure synchronized exposure, cells were allowed to adhere for 6 hours under normoxic conditions (37°C, 21% O₂, 5% CO₂). Subsequently, the medium was replaced with fresh, prewarmed medium to remove any residual trypsin effects. For the normoxic control group, cells were continuously cultured in a standard incubator (37°C, 21% O₂, 5% CO₂). For the hypoxic group, the culture plates were immediately transferred to a Trigas incubator (Heal Force) and exposed to a gas mixture of 1% O₂, 5% CO₂, balanced with N₂. The oxygen concentration was monitored and stably maintained at 1% throughout the experiment. Cells were subjected to hypoxic conditions for 24 hours before subsequent analysis.

### Gene knockdown

Small interfering RNAs (siRNAs) targeting HIF-1α and NC (negative control) were purchased from Genepharma and then transfected into MCCs using Lipofectamine RNAiMAX reagent (Invitrogen), following the manufacturer’s protocol. The sequences of siRNA1 were as follows: the sense strand was 5′-AGUUGGAACUGGUGGAAAATT-3′, and the antisense strand was 5′-UUUUCCACCAGUUCCAACUTT-3′. The sequences of siRNA2 were as follows: the sense strand was 5′-UGGAAUCUGAGGACACGAGTT-3′, and the antisense strand was 5′-CUCGUGUCCUCAGAUUCCATT-3′. Transfection efficiency was confirmed through real-time qPCR and Western blotting.

### RNA extraction and quantitative reverse transcription PCR analysis

Total RNA was extracted from MCCs using AG RNAex Pro reagent (Accurate Biotechnology) according to the manufacturer’s protocol. Reverse transcription was performed with 1 µg total RNA using the Hifair III 1st Strand cDNA Synthesis SuperMix for qPCR (Yeasen Biotech). Quantitative real-time PCR was conducted with Hieff qPCR SYBR Green Master Mix (Yeasen Biotech) on a thermal cycler (Applied Biosystems). Relative gene expression was calculated using the 2⁻ΔΔCt method. All primer sequences are listed in Table 1.

**Table 1:** Primers used for RT-qPCR.

Gene		Primer sequence (5′-3′)
*Tuba1a*	F	CTGGCTTCAAGGTTGGCATTA
*Tuba1a*	R	CACGCTTGGCATACATCAGAT
*Mmp13*	F	TGATGCTAACCAGACTATGGACAA
*Mmp13*	R	ATGACTCTCACAATGCGATTACTC
*Adamts5*	F	GGTCAGTGTTCTCGCTCTTG
*Adamts5*	R	GTTAGGTGGGCAGGGTATGA
*Mmp3*	F	ATGAACTTGGCCACTCCCTG
*Mmp3*	R	GTGGGTACCACGAGGACATC
*Il6*	F	ACAAGTCCGGAGAGGAGACT
*Il6*	R	GAATTGCCATTGCACAACTCT
*Tnf*	F	ATGGGCTCCCTCTCATCAGT
*Tnf*	R	GCTTGGTGGTTTGCTACGAC
*Nos2*	F	CCTCCTTGTTCAACTCACCTTC
*Nos2*	R	CAATCCACAACTCGCTCCAA
*Tp53*	F	ATGACTGAGGTCGTGAGACG
*Tp53*	R	CTCATACGGTACCACCACGC
*Mdm2*	F	CTTCGTGAGAACTGGCTTCC
*Mdm2*	R	ACTCCTTAGCATCATCCTCTGT

### Western blotting

Cells were lysed in RIPA buffer (Beyotime) supplemented with a protease inhibitor cocktail (Roche, Switzerland). Protein concentration was determined using the BCA assay (Beyotime). Equal amounts of protein were separated by SDS-PAGE and transferred onto PVDF membranes (Millipore). The membranes were blocked with 5% nonfat milk and incubated with the following primary antibodies: HIF-1α (1:1000; Abcam), p53 (1:1000; ABclonal), TNF-α (1:1000; Huabio), MMP13 (1:1000; Abcam), iNOS (1:1000; Servicebio), BAX (1:1000; Proteintech), p21 (1:1000; Abcam), MDM2 (1:1000; Immunoway), and α-Tubulin (1:5000; Servicebio). After incubation with HRP-conjugated secondary antibodies (1:5000; Jackson ImmunoResearch), signals were visualized using an enhanced chemiluminescence kit (Thermo Fisher Scientific). The band intensities were quantified using ImageJ software (National Institutes of Health) and normalized to α-tubulin.

### Immunofluorescence staining

MCCs were seeded on glass coverslips and cultured to 60% to 70% confluence. After treatment, cells were fixed with 4% paraformaldehyde, permeabilized with 0.1% Triton X-100, and blocked with 5% BSA. Cells were then incubated with primary antibody against IL-6 (1:500; Zenbio) and MMP3 (1:1000; Servicebio) overnight at 4°C, followed by Alexa Fluor-conjugated secondary antibody (1:500, Invitrogen) for 1 hour at room temperature. Negative controls were included by omitting primary antibodies and incubating cells with PBS only during each antibody incubation step to ensure specific staining (Figure S1A). Nuclei were counterstained with DAPI. Images were acquired using a confocal microscope (Leica Microsystems) and analysed with ImageJ.

### Differential expression and enrichment analysis

Raw gene counts were quantified using RSEM. Differential expression analysis between the DMOG-treated (1 mM) and control groups under hypoxic conditions was performed using DESeq2. Genes with |log2FC| ≥ 1 (indicating a 2-fold change) and adjusted *P* value (FDR) <.05 were defined as differentially expressed genes. Gene Ontology and Kyoto Encyclopedia of Genes and Genomes pathway enrichment analyses of the differentially expressed genes were performed using clusterProfiler, with terms/pathways having a Bonferroni-corrected *P* value <.05 considered significantly enriched.

### Coimmunoprecipitation (Co-IP) assay

Hypoxic condylar chondrocytes were treated with or without 1 mM DMOG for 24 hours under 1% O₂. Cells were lysed in ice-cold IP lysis buffer (50 mM Tris–HCl pH 7.4, 150 mM NaCl, 1 mM EDTA, 1% NP-40, 10% glycerol, 1 mM Na₃VO₄, 1 mM PMSF, and protease inhibitor cocktail). Lysates were cleared by centrifugation (12,000 × *g*, 15 minutes, 4°C), and protein concentration was determined using a BCA kit. Equal amounts of protein (500-800 μg) were incubated overnight at 4°C with anti-p53 antibody (or anti-MDM2 antibody) or normal rabbit/mouse IgG as a negative control. Subsequently, Protein A/G Magnetic Beads (MCE, HY-K0202) were added and incubated for 2 hours at 4°C with rotation. The magnetic bead complexes were washed five times with cold IP wash buffer (50 mM Tris–HCl pH 7.4, 300 mM NaCl, 0.1% NP-40) using a magnetic separator. Bound proteins were eluted by boiling in 2× SDS-PAGE loading buffer. Eluates were resolved by SDS-PAGE and analysed by Western blotting using anti-MDM2, antiubiquitin, or anti-p53 antibodies, respectively. Input (5% of total lysate) was included as a loading control.

### Chromatin immunoprecipitation (ChIP) assay

Hypoxic condylar chondrocytes were treated with or without 1 mM DMOG for 24 hours under 1% O₂. Cross‑linking was performed with 1% formaldehyde (10 minutes, RT) and quenched with 0.125 M glycine. Cells were scraped, pelleted, and lysed in ChIP lysis buffer (50 mM Tris–HCl pH 8.0, 10 mM EDTA, 1% SDS, 1 mM PMSF, protease inhibitors). Chromatin was sheared to 200 to 500 bp by sonication (Bioruptor, 30 seconds ON/30 seconds OFF cycles, 10-12 cycles, 4°C). Sonicated chromatin was diluted 10‑fold in ChIP dilution buffer (16.7 mM Tris–HCl pH 8.0, 167 mM NaCl, 1.2 mM EDTA, 1% Triton X‑100, 0.01% SDS) and pre‑cleared with Protein A/G Magnetic Beads (MCE, HY-K0202) for 1 hour at 4°C. An aliquot (1%) was saved as Input. The remainder was incubated overnight at 4°C with anti‑HIF‑1α antibody or normal rabbit IgG. Protein A/G Magnetic Beads were then added and incubated for 2 hours at 4°C. The beads were washed sequentially with low‑salt, high‑salt, LiCl, and TE buffers using a magnetic separator. Complexes were eluted in ChIP elution buffer (1% SDS, 0.1 M NaHCO₃). Cross‑links were reversed by incubation at 65°C overnight, followed by proteinase K digestion. DNA was purified using a PCR purification kit (Qiagen). Enrichment of HIF‑1α‑bound *Mdm2* promoter and the positive control *Vegfa* promoter was quantified by qPCR using SYBR Green Master Mix. Primers flanking the predicted HIF‑1α binding site (HRE) in the rat *Mdm2* promoter and the well‑characterized HRE in the *Vegfa* promoter were used; the sequences are listed in Table 2. Results are presented as %Input after normalization to IgG control.

**Table 2:** Primers used for ChIP.

Gene		Primer sequence (5′-3′)
*Mdm2*	F	GGCCATGATGCCCTTATTCA
*Mdm2*	R	CTCTCCCCTCCCTCCATTAC
*Vegfa*	F	ACGGGGAGATCGTGAACTTG
*Vegfa*	R	TCACAAACACACTATACCCAGACA

### Statistical analysis

All statistical analyses were performed using GraphPad Prism software (v.8.0.1; GraphPad Software). Data are presented as the mean ± standard deviation. An independent-samples *t* test was used to compare differences between two groups, while one-way analysis of variance (ANOVA) followed by Tukey’s post hoc test was used for multiple group comparisons. A *P* value <.05 was considered statistically significant. All in vitro experiments were repeated at least three times independently (biological replicates), and in vivo experiments included *n* = 6 animals per group.

## Results

### UAC triggers early condylar degeneration and a hypoxic microenvironment in rats

**Fig. 1:**
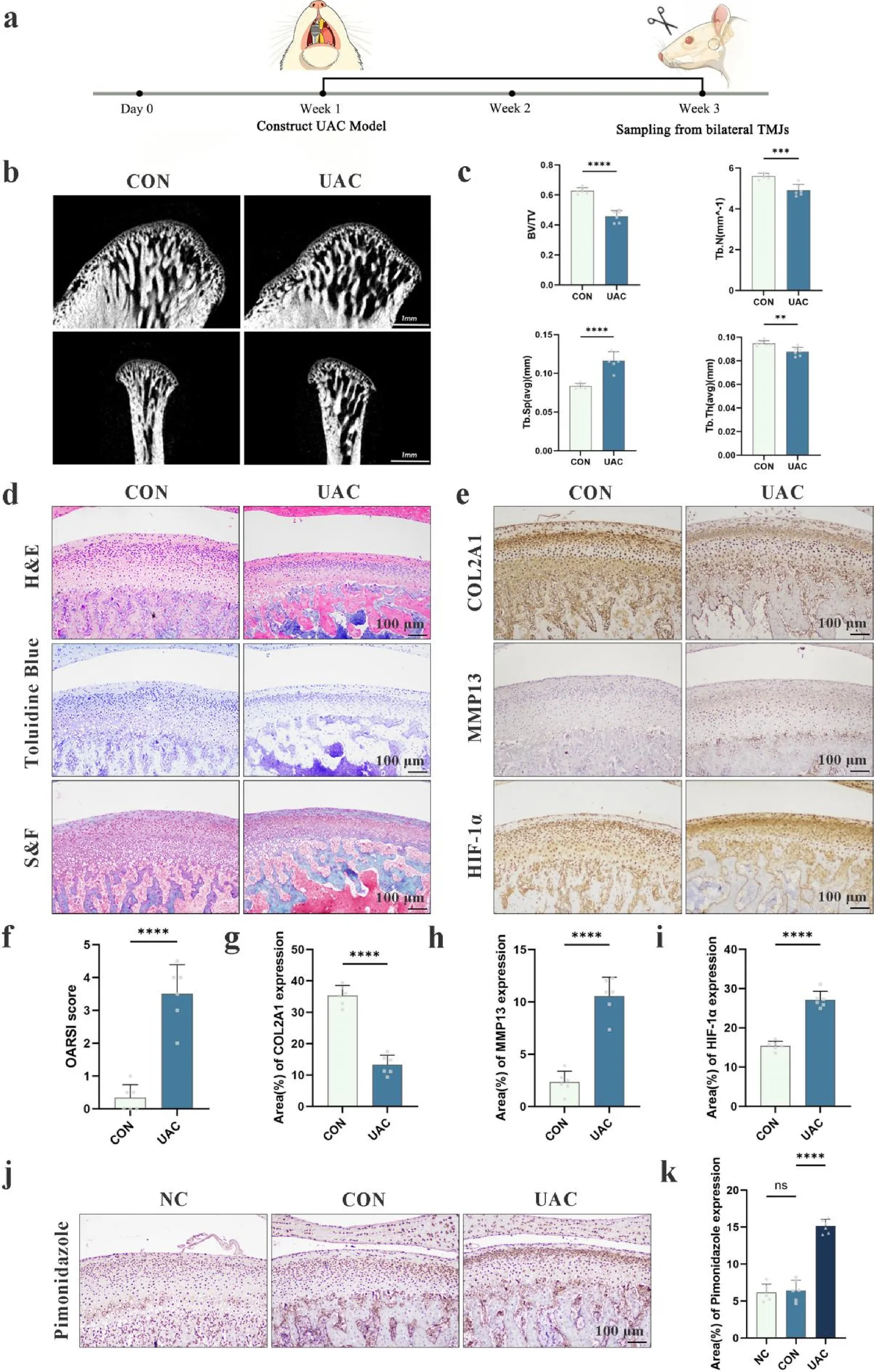
UAC triggers early condylar degeneration and a hypoxic microenvironment in rats. (A) Timeline of the UAC model construction and sample collection. (B) Representative micro-CT images of the subchondral bone microstructure from the control and UAC groups. Scale bar: 1 mm. (C) Quantitative analysis of subchondral bone parameters based on micro-CT images. (D) Representative images of H&E staining, toluidine blue staining, and S&F staining of rat condyles from the control and UAC groups at the 2-week time point. Scale bar: 100 μm. (E) Representative immunohistochemical images of COL2A1, MMP13, and HIF-1α in condylar chondrocytes. Scale bar: 100 μm. (F) OARSI pathological score based on S&F staining. (F-H) Quantitative analysis of the immunohistochemical staining intensity of COL2A1 (G), MMP13 (H), and HIF-1α (I). (J) Representative immunohistochemical images of pimonidazole adducts (hypoxia probe) in condylar chondrocytes. Scale bar: 100 μm. (K) Quantitative analysis of pimonidazole-positive area. Data are presented as mean ± SEM (*n* = 6 per group). Statistical significance between the control and UAC groups was determined by unpaired two-tailed Student’s *t* test (or one-way ANOVA if more than two groups are compared in this figure). ns, no significance, **P < .01, ***P < .001, ****P < .0001.

To explore the early-stage alterations in TMJOA, a 2-week UAC model was established in rats (Figure 1A). Micro-CT image analysis indicated that compared with the control group, BV/TV, Tb.N and Tb.Th in UAC group were significantly decreased, while Tb.Sp was significantly increased (Figure 1B, C). The integrated use of various staining techniques, including H&E, Toluidine Blue, and S&F staining, revealed that in the TMJOA experimental group, the thickness of the condylar cartilage layer was significantly reduced, and the number of chondrocytes also decreased markedly (Figure 1D). Furthermore, multiple layers of the condylar cartilage, such as the fibrous layer, hypertrophic layer, and proliferative layer, exhibited varying degrees of thinning (Figure 1D). Additionally, significant differences in functional properties were observed between the UAC and control groups, as evidenced by the Osteoarthritis Research Society International (OARSI) scores (Figure 1F). Immunohistochemical analysis indicated that the expression level of COL2Α1 was notably downregulated, whereas that of MMP13 was significantly upregulated, suggesting abnormal molecular regulation during the pathological process of TMJOA (Figure 1E, G, H). Meanwhile, we found that the expression of HIF-1α was also increased, which suggests that a hypoxic environment is generated in the early stage of TMJOA (Figure 1E, I). The hypoxia probe further corroborated this observation. We discovered that there was no notable disparity in the immunohistochemistry of pimonidazole in the condyle between the negative control rats injected with PBS and the control group rats injected with the hypoxia probe; conversely, the expression of pimonidazole in the condyle of UAC group was significantly elevated (Figure 1J, K), which suggests that there is a presence of a hypoxic microenvironment in the initial stages of TMJOA, as opposed to the normal joint cartilage. These findings confirm that early TMJOA is characterized by concurrent cartilage degeneration and the formation of a hypoxic niche, which may drive disease progression.

### Hypoxic environment induces inflammatory response and matrix degradation in chondrocytes

**Fig. 2:**
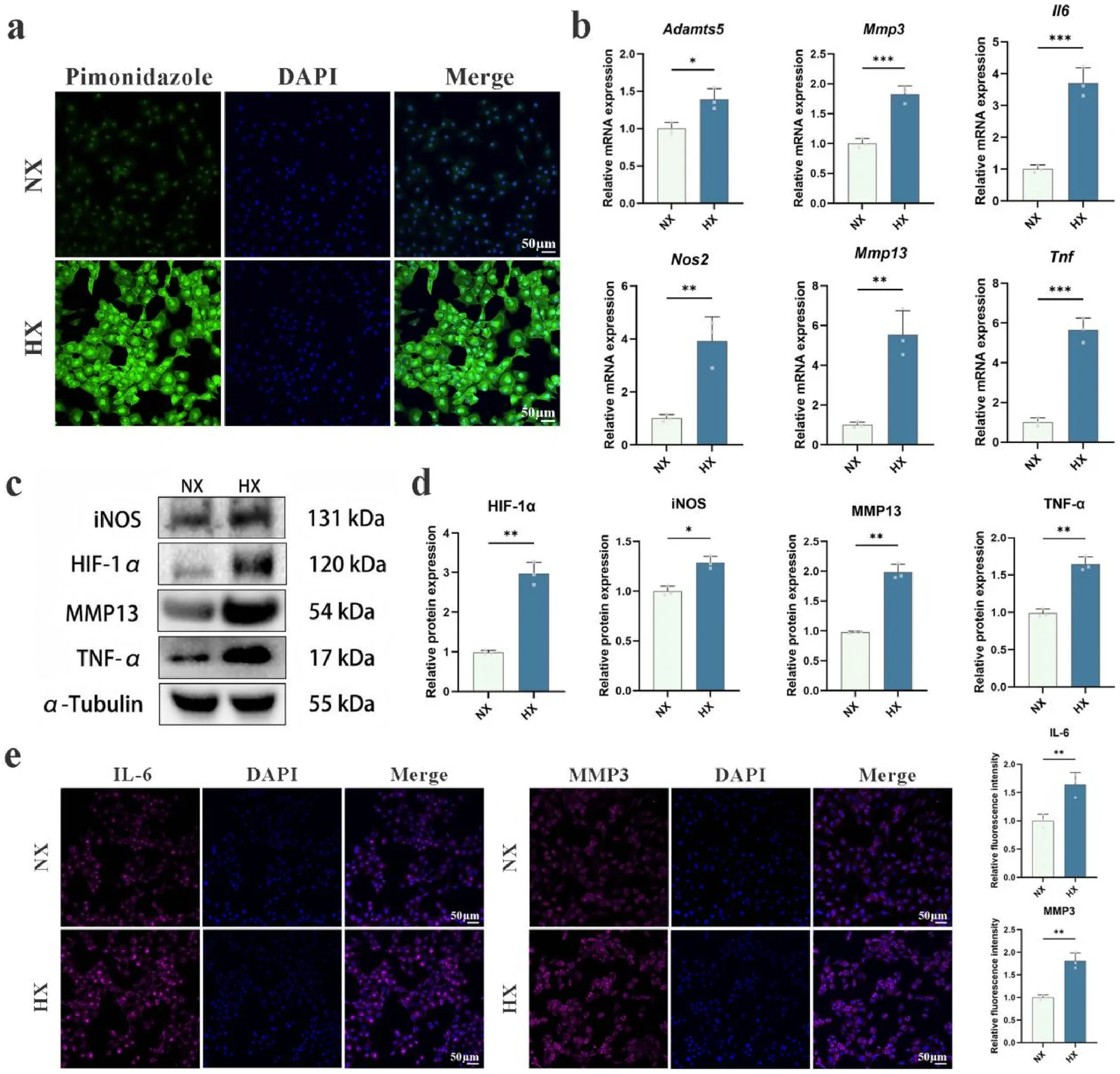
Hypoxic conditions (1% O₂ for 24 hours) induce an inflammatory response and matrix degradation in chondrocytes. (A) Image of immunofluorescence staining of pimonidazole in chondrocytes under normoxia (NX) and hypoxia (HX) for 24 hours. Scale bar: 50 μm. (B) Relative mRNA expression levels of *Adamts5, Mmp3, Il6, Nos2, Mmp3*, and *Tnf* in chondrocytes treated with hypoxia. (C and D) Western blot and quantitative analysis of HIF-1α, iNOS, MMP13, and TNF-α protein levels in chondrocytes after 24 hours of hypoxia, normalized to α-Tubulin and expressed relative to the NX group. (E) Representative immunofluorescence images and quantitative analysis of IL-6 and MMP3 in chondrocytes treated with hypoxia. Scale bar: 50 μm. Data are presented as mean ± SEM (*n* = 3 per group). Statistical significance was determined by unpaired Student’s *t* test. *P < .05, **P < .01, ***P < .001.

To investigate whether hypoxia triggers an inflammatory response in chondrocytes, cells were cultured at 1% O₂ for 24 hours to mimic a pathological hypoxic environment. The efficacy of the hypoxic intervention was verified by immunofluorescence staining using the hypoxia probe pimonidazole (Figure 2A, Figure S1D). We first ruled out cytotoxicity as a confounding factor for subsequent inflammatory and matrix-related measurements. The CCK-8 assay showed no significant change in chondrocyte viability between normoxia and hypoxia groups (Figure S1E). Consistent with this result, Annexin V/PI flow cytometry detected no statistically significant difference in the total apoptotic cell proportion under normoxic or hypoxic culture (Figure S1C), indicating that 24 hours of short-term hypoxia does not induce obvious chondrocyte death. We further evaluated the synthesis capacity of chondrocyte extracellular matrix under sustained hypoxic stimulation via toluidine blue staining after 7 days of culture. The staining intensity of sulphated glycosaminoglycans, the major component of cartilage matrix, was markedly reduced relative to the normoxic group (Figure S1F), which visually confirmed that prolonged hypoxia suppressed extracellular matrix deposition by chondrocytes. The results of qRT-PCR demonstrated that the expression of *Adamts5, Mmp3, Il6, Nos2, Mmp13*, and *Tnf* was significantly upregulated following 24 hours of hypoxia intervention (Figure 2B). The results of Western blot analysis also revealed that the expressions of iNOS, MMP13, and TNF-α were significantly elevated after 24 hours of hypoxia intervention (Figure 2C, D). In line with the above results, immunofluorescence staining showed that hypoxia significantly enhanced the fluorescence intensity of IL-6 and MMP3 in chondrocytes (Figure 2E).

In summary, our data demonstrate that hypoxia independently drives chondrocyte inflammation and matrix breakdown without causing cell death. Mechanistically, short-term hypoxia elevates proinflammatory factors and cartilage-degrading enzymes, while prolonged hypoxic stimulation directly inhibits extracellular matrix synthesis, jointly contributing to irreversible matrix degradation in chondrocytes.

### HIF-1α stabilization suppresses hypoxia-induced inflammatory response and matrix degradation in condylar cartilage

**Fig. 3:**
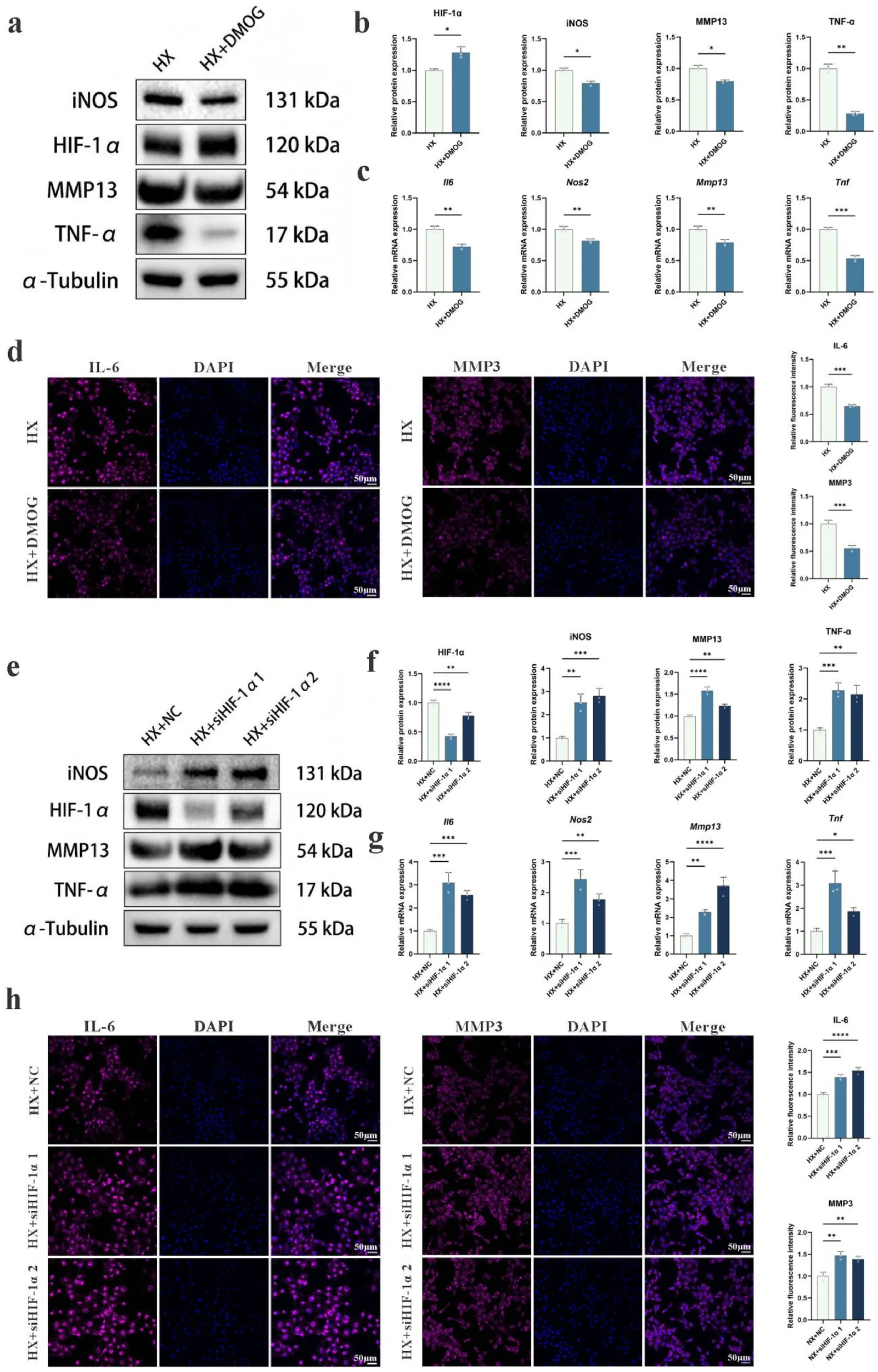
HIF-1α stabilization suppresses hypoxia-induced inflammatory response and matrix degradation in condylar cartilage. (A and B) Western blot images and quantitative analysis of HIF-1α, iNOS, MMP13, and TNF-α protein levels in chondrocytes under hypoxia and hypoxia + DMOG (HX + DMOG) conditions. (C) Relative mRNA expression levels of *Il6, Nos2, Mmp13*, and *Tnf* in chondrocytes treated with DMOG under hypoxia, normalized to the HX group. (D) Representative immunofluorescence images and quantitative analysis of IL-6 and MMP3 in chondrocytes under hypoxia and hypoxia + DMOG conditions. Scale bar: 50 μm. (E and F) Western blot images and quantitative analysis of HIF-1α, iNOS, MMP13, and TNF-α protein levels in chondrocytes under hypoxia and hypoxia with transfection of NC or two different HIF-1α-specific siRNAs (siHIF-1α 1, siHIF-1α 2). (G) Relative mRNA expression levels of *Il6, Nos2, Mmp13*, and *Tnf* in chondrocytes transfected with HIF-1α-specific siRNA or NC under hypoxia. (H) Representative immunofluorescence images and quantitative analysis of IL-6 and MMP3 in chondrocytes transfected with HIF-1α-specific siRNA or NC under hypoxia. Scale bar: 50 μm. Data are presented as mean ± SEM (*n* = 3 per group). Statistical significance was determined by unpaired Student’s *t* test for comparisons between two groups (panels A-D), or by one-way analysis of variance (ANOVA) followed by Tukey’s post hoc test for comparisons among three or more groups (panels E-H). *P < .05, **P < .01, ***P < .001, ****P < .0001.

Previous research has demonstrated that articular cartilage, as an avascular tissue, maintains a physiological hypoxic microenvironment. This microenvironment upholds chondrocyte homeostasis through HIF-1α.^34^ In order to investigate the function of HIF-1α within the hypoxic microenvironment of condylar cartilage, this study employed the prolyl hydroxylase (PHD) inhibitor DMOG (1 mM) to stabilize HIF-1α. The results of Western blot analyses demonstrated that the expression levels of iNOS, MMP13, and TNF-α experienced a significant reduction subsequent to DMOG administration under hypoxic conditions, and qRT-PCR yielded consistent trends (Figure 3A-C). Fluorescence staining for IL-6 and MMP3 in the chondrocytes revealed that the intensity of both IL-6 and MMP3 was significantly reduced following DMOG treatment under hypoxic conditions (Figure 3D). This indicates that in the hypoxic microenvironment of condylar cartilage, enhancing HIF‑1α stability can counteract inhibit the expression of inflammatory factors and matrix-degrading enzymes. Moreover, the observation that DMOG treatment similarly downregulated inflammatory and catabolic markers in chondrocytes under normoxic conditions suggests that its chondroprotective action may not strictly depend on a pre-existing hypoxic milieu, but rather hinges on the pharmacological stabilization of HIF-1α protein itself (Figure S2A-D).

To further verify whether HIF-1α plays a protective role in hypoxia-induced osteoarthritis, we utilized siRNA to knock down HIF-1α under hypoxic conditions and observed its effect on chondrocytes. The mRNA expression levels of *Il6, Nos2, Mmp13*, and *Tnf*, as well as the protein expression levels of iNOS, MMP13, and TNF-α, were further elevated upon HIF-1α knockdown (Figure 3E-G). Fluorescence staining of IL-6 and MMP3 in chondrocytes demonstrated that the intensity of IL-6 and MMP3 was markedly enhanced subsequent to HIF-1α knockdown under hypoxic conditions (Figure 3H). The findings suggest that knockdown of the HIF-1α gene can exacerbate the hypoxia-induced inflammatory response and matrix degradation in chondrocytes. In contrast, knockdown of HIF-1α under normoxic conditions did not cause significant alterations in the expression of these inflammatory and catabolic markers compared to the normoxic control (Figure S2E-H). These results demonstrate that HIF‑1α acts as a negative regulator of the inflammatory and matrix-degrading response induced by severe hypoxia in condylar chondrocytes. The lack of effect under normoxia is likely attributable to the low basal expression level of HIF-1α in this condition, wherein its knockdown exerts a minimal impact on cellular homeostasis.

### DMOG ameliorated OA development in UAC rat model

**Fig. 4:**
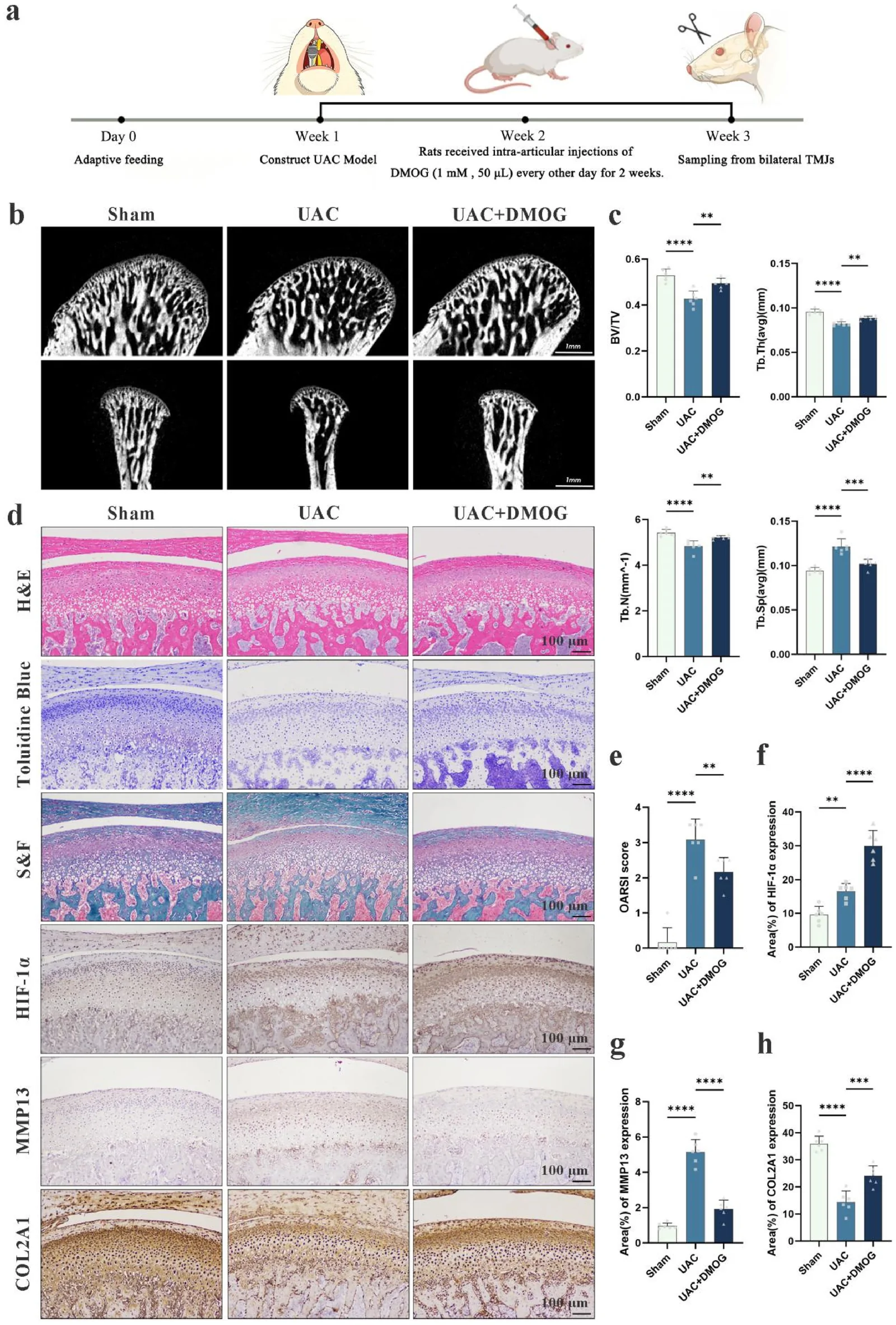
DMOG mitigated the progression of TMJOA in UAC rat model. (A) Timeline of the UAC model construction, DMOG intervention, and sample collection. (B) Representative micro-CT images showing the subchondral bone microstructure in Sham, UAC, and UAC + DMOG groups. Scale bar: 1 mm. (C) Quantitative analysis of subchondral bone parameters (BV/TV, Tb.N, Tb.Sp, Tb.Th) derived from micro-CT images. (D) Representative images of H&E staining, toluidine blue staining, S&F staining, and immunohistochemical staining for HIF-1α, MMP13, and COL2A1. Scale bar: 100 μm. (E) TMJOA pathological scores (OARSI score) based on S&F staining. (F) Quantitative analysis of HIF-1α expression. (G) Quantitative analysis of MMP13 expression. (H) Quantitative analysis of COL2A1 expression. Data are presented as mean ± SEM (*n* = 6 per group). Statistical significance was determined by one-way ANOVA followed by Tukey’s post hoc test. **P < .01, ***P < .001, ****P < .0001.

To assess the therapeutic potential of HIF‑1α stabilization in vivo, DMOG (1 mM) or vehicle was injected into the temporomandibular joints of UAC‑model rats every other day for 2 weeks (Figure 4A). Micro-CT analysis revealed that DMOG restored the subchondral bone microstructure of the TMJ in TMJOA rats (Figure 4B, C). Histological evaluation of cartilage integrity through H&E, Toluidine Blue, and S&F staining indicated that treatment with UAC combined with DMOG mitigated these degenerative changes (Figure 4D). Consistent with the results of histological staining, DMOG decreased the OARSI score in the UAC group (Figure 4E). Immunohistochemical staining revealed that DMOG upregulated the expression of HIF-1α and COL2Α1 in the condylar cartilage layer while inhibited the protein expression of MMP13 in UAC-induced TMJOA (Figure 4D-H). TUNEL staining revealed no significant difference in chondrocyte apoptosis between the UAC and UAC + DMOG groups (Figure S3A), indicating that DMOG-mediated cartilage protection is likely achieved through suppressing catabolic and inflammatory responses rather than inhibiting apoptosis. Collectively, these findings demonstrate that stabilizing HIF-1α in vivo effectively attenuates TMJOA progression by mitigating inflammation and matrix degradation.

### DMOG-mediated HIF-1α stabilization exerts a protective effect by inhibiting p53 signalling transduction

**Fig. 5:**
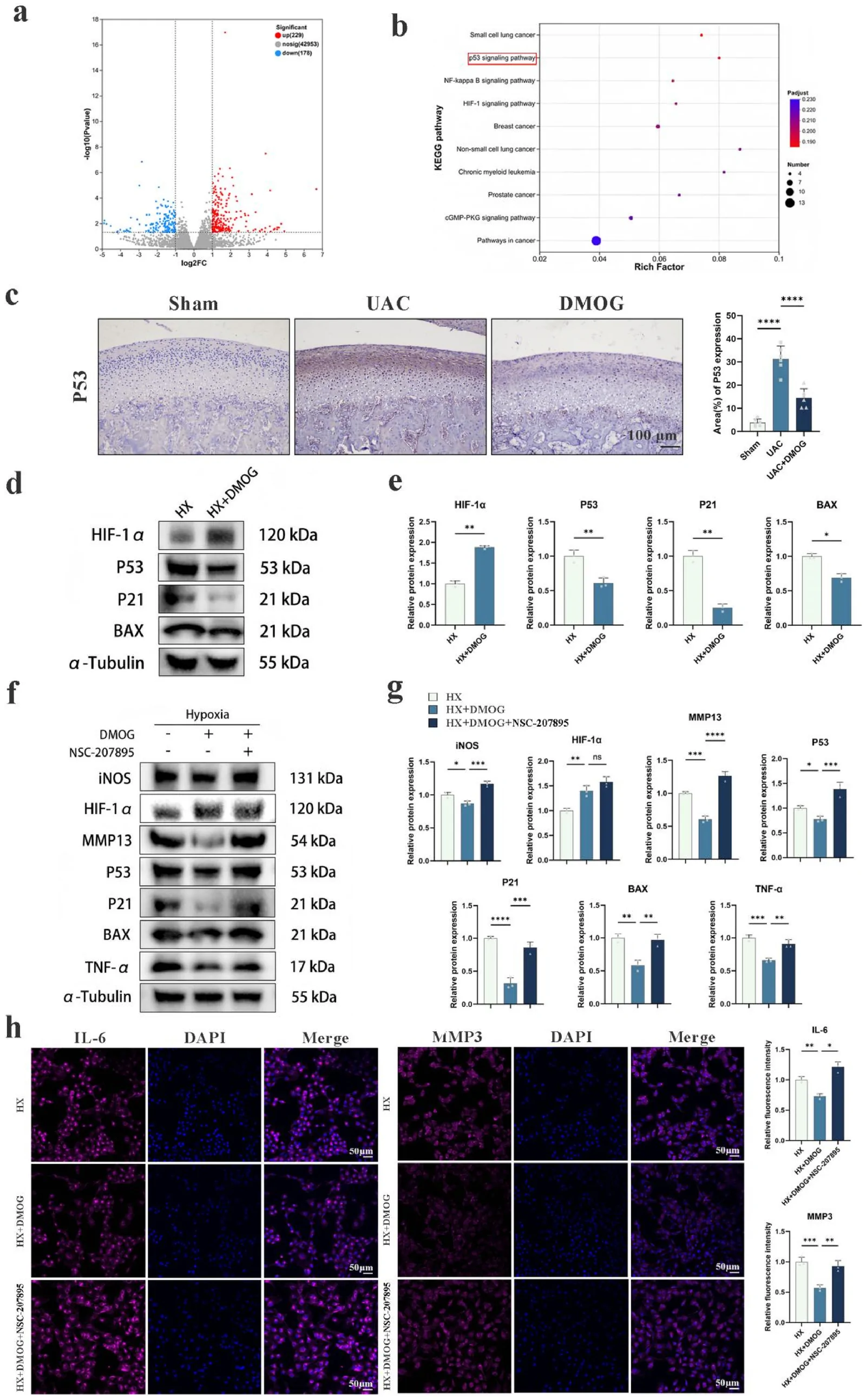
DMOG-mediated HIF-1α stabilization exerts a protective effect by inhibiting p53 signalling transduction. (A) Volcano plot of DEGs in chondrocytes treated with or without DMOG (*P* < .05, |fold change| > 2) under hypoxic conditions. (B) KEGG pathway enrichment analysis of DEGs. (C) Representative immunohistochemical images of p53 in condylar chondrocytes (scale bar: 100 μm) and quantitative analysis of p53-positive area (*n* = 6). (D and E) Western blot and quantitative analysis of HIF-1α, p53, p21, and BAX protein levels in chondrocytes under hypoxia and hypoxia + DMOG conditions. (F and G) Western blot images and quantitative analysis of iNOS, HIF-1α, MMP13, p53, p21, BAX, and TNF-α protein levels in chondrocytes under hypoxia, hypoxia + DMOG, and hypoxia + DMOG + NSC-207895 (HX + DMOG + NSC-207895) conditions (*n* = 3). (H) Immunofluorescence staining images and quantitative analysis of IL-6 and MMP3 in chondrocytes treated with DMOG and NSC-207895 under hypoxia (*n* = 3). Scale bar: 50 μm. Data are presented as mean ± SEM. Statistical significance was determined by unpaired Student’s *t* test (for pairwise comparisons) or one-way ANOVA (for multigroup comparisons), as appropriate. ns, no significance, *P < .05, **P < .01, ***P < .001, ****P < .0001.

To further determine the effects of DMOG on targeted genes within chondrocytes, the RNA sequencing analysis was conducted on chondrocytes subjected to hypoxic conditions, with or without DMOG treatment. Overall, 229 genes were significantly upregulated, while 178 were significantly downregulated in the hypoxia with DMOG-treatment group compared with hypoxia group (Figure 5A). Kyoto Encyclopedia of Genes and Genomes analysis revealed significant enrichment of p53 signalling pathway (Figure 5B). Immunohistochemical staining of p53 in rat TMJ cartilage sections further confirmed that DMOG treatment significantly reduced p53 protein expression in situ (Figure 5C). Based on the above, we conducted Western blot and verified that stable HIF-1α significantly downregulated the p53 pathway under hypoxic conditions (Figure 5D, E).

To functionally test whether p53 inhibition contributes to the protective effect of HIF‑1α stabilization, we employed the p53 agonist NSC‑207895 (0.5 μM). Co‑treatment with NSC‑207895 effectively reversed the DMOG‑induced downregulation of p53, p21, BAX, iNOS, MMP13, and TNF‑α at the protein level (Figure 5F, G). Further immunofluorescence confirmed that the p53 activator NSC-207895 reversed the downregulation of IL-6 and MMP3 induced by DMOG (Figure 5H). These results suggest that DMOG alleviates the inflammatory response and matrix degradation of chondrocytes under hypoxia by inhibiting the p53 signalling pathway through the stabilization of HIF-1α.

### HIF-1α directly transactivates *Mdm2* to promote p53 ubiquitin-proteasomal degradation

**Fig. 6:**
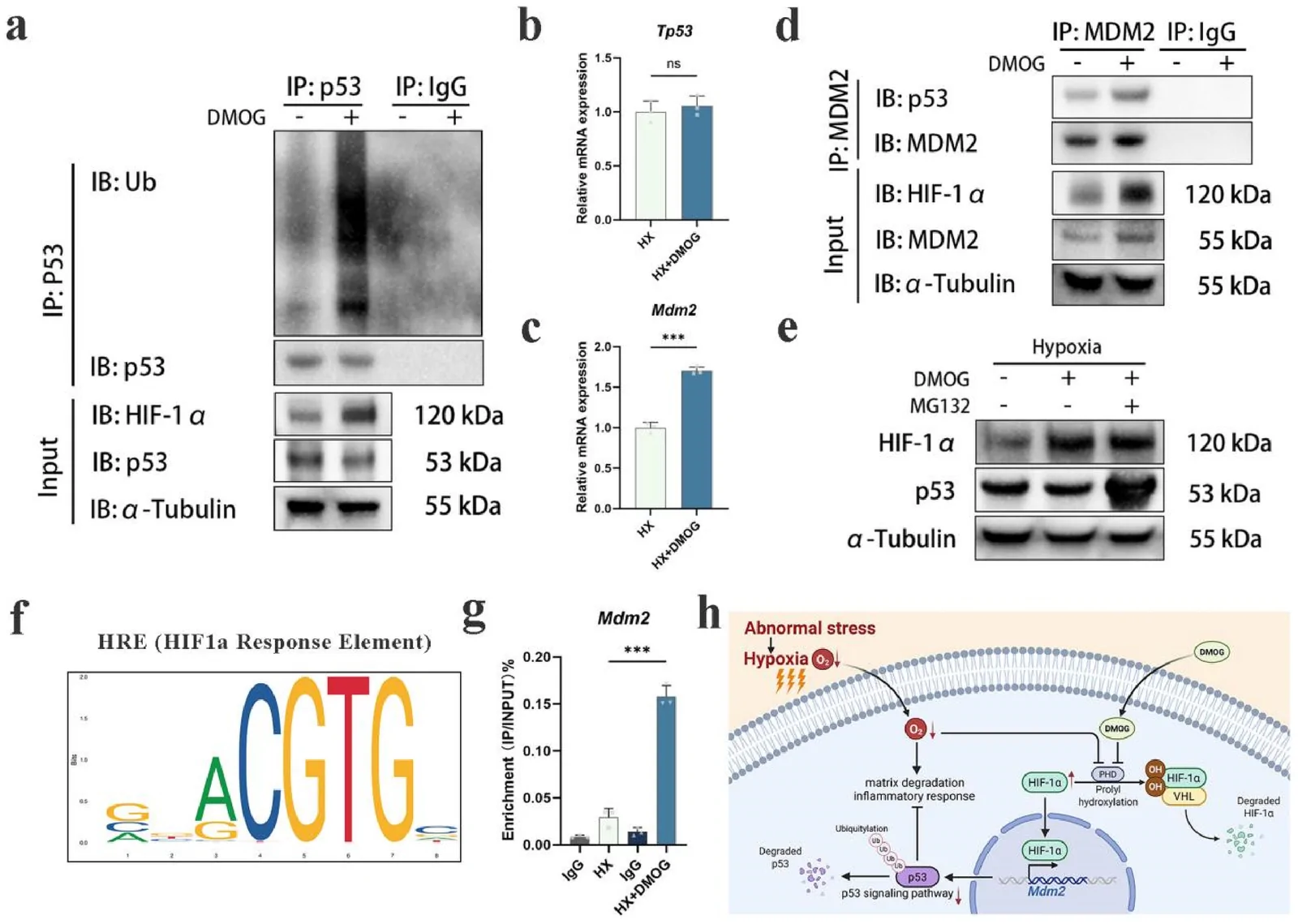
Stabilized HIF-1α transcriptionally activates *Mdm2* to promote p53 ubiquitin-proteasomal degradation in hypoxic condylar. (A) Co-IP assay detecting endogenous p53 ubiquitination in hypoxic chondrocytes treated with or without 1 mM DMOG. (B and C) qRT-PCR analysis of *Tp53* and *Mdm2* mRNA expression in chondrocytes cultured under hypoxia with or without DMOG intervention. (D) Reciprocal Co-IP assay for detecting physical interaction between MDM2 and p53 in hypoxic chondrocytes with or without DMOG treatment. (E) Western blot analysis of p53 protein abundance in hypoxic chondrocytes incubated with DMOG in the presence or absence of 10 μM MG132. (F) JASPAR database-based in silico prediction of potential HIF-1α binding sites on rat *Mdm2* promoter. (G) ChIP-qPCR assay measuring HIF-1α occupancy on the *Mdm2* promoter in hypoxic chondrocytes with or without DMOG. (H) Schematic diagram. Data are presented as mean ± SEM (*n* = 3 per group). Statistical significance was determined by unpaired Student’s *t* test. **P < .01, ***P < .001.

In the above functional experiments, we confirmed that DMOG-mediated HIF-1α stabilization markedly repressed p53 signalling activity in hypoxic condylar chondrocytes. To elucidate the underlying molecular mechanism, we first assessed the transcriptional level of *Tp53*. qRT-PCR analysis revealed no significant difference in *Tp53* mRNA abundance between hypoxic chondrocytes with or without DMOG intervention (Figure 6B), indicating that HIF-1α suppresses p53 expression primarily through post-translational regulation.

Given that ubiquitination-dependent proteasomal degradation is the core machinery governing p53 turnover,^35^ we hypothesized that stabilized HIF-1α accelerates p53 degradation. Co-IP assays targeting endogenous p53 validated this conjecture (Figure 6A). Under hypoxic culture, DMOG treatment robustly elevated the ubiquitination level of immunoprecipitated p53, concomitant with a reduction in total p53 protein abundance, confirming that HIF-1α stabilization facilitates p53 ubiquitin modification.

To identify the E3 ubiquitin ligase responsible for this effect, we focused on MDM2, the canonical E3 ligase mediating p53 ubiquitination and degradation.^36^^,^^37^ We therefore examined MDM2 expression and found that DMOG significantly upregulated both *Mdm2* mRNA and protein levels (Figure 6C). Reciprocal Co-IP with anti-MDM2 antibody further demonstrated that MDM2 physically interacted with p53, and that this interaction was markedly strengthened by DMOG-induced HIF-1α accumulation (Figure 6D).

To verify that DMOG reduces p53 abundance via ubiquitin-proteasome-dependent degradation, we incubated hypoxic chondrocytes with the proteasome inhibitor MG132 (10 μM). Western blot assays revealed that MG132 fully abolished DMOG-induced p53 reduction and rescued p53 protein stability (Figure 6E). Given that HIF-1α functions as a transcriptional activator under hypoxia, we reasoned that HIF-1α might directly bind to these sites to upregulate *Mdm2* expression,^38^ we reasoned that HIF-1α might directly bind to the *Mdm2* promoter to upregulate its expression. In silico analysis via the JASPAR database subsequently predicted putative HIF-1α binding sites within the rat *Mdm2* promoter (Figure 6F). Chromatin ChIP-qPCR assays further demonstrated that HIF-1α stabilization by DMOG markedly enhances HIF-1α occupancy on the Mdm2 promoter fragment (Figure 6G). The canonical HIF-1α target gene *Vegfa* was simultaneously detected as a positive control to ensure the validity of our ChIP-qPCR experimental system (Figure S3B).

Collectively, these data elucidate a complete regulatory axis: stabilized HIF-1α directly binds the *Mdm2* promoter to transcriptionally activate MDM2 expression; augmented MDM2 protein then promotes robust p53 ubiquitination and proteasomal degradation, thereby restraining the procatabolic and proinflammatory p53 signalling pathway in hypoxic condylar chondrocytes (Figure 6H).

## Discussion

Our research indicates that degenerative changes in the TMJ occur as early as 2 weeks following the induction of UAC, characterized by reduced cartilage thickness, a decreased number of chondrocytes, and alterations in the subchondral bone microstructure. Furthermore, a hypoxic microenvironment is present in the condylar cartilage during the early stage of TMJOA. In vitro experiments have revealed that pathological hypoxia amplifies the inflammatory response and matrix degradation in cartilage. Nevertheless, stabilizing HIF-1α under hypoxic conditions exerts a protective effect via transcriptional upregulation of *Mdm2* and subsequent p53 signalling suppression, thereby mitigating the inflammatory response and matrix degradation in condylar chondrocytes.

Most previous studies have proven the existence of a hypoxic environment in OA using hypoxia markers such as HIF-1α.^39^ Studies have revealed that the upregulation of HIF-1α in OA is mainly driven by nonhypoxic factors such as inflammation, oxidative stress, mechanical stress, and metabolic abnormalities.^40^, ^41^, ^42^ Hypoxia itself can promote cartilage catabolism, but HIF-1α activation acts as a negative feedback mechanism to counteract this damage. The innovative application of hypoxia probes in this experiment further verified the existence of a hypoxic microenvironment in the early stage of TMJOA. Articular cartilage is naturally avascular and physiologically hypoxic; however, pathological hypoxia exacerbates cartilage degeneration.^43^ These findings underscore hypoxia as a potent proinflammatory stimulus in TMJ chondrocytes, establishing a link between environmental stress and molecular inflammation. Recent studies in related oral and craniofacial tissues have similarly highlighted the interplay between microenvironmental stress and inflammatory cascades. For example, MZB1 was shown to exacerbate periodontitis by promoting ER stress-mediated inflammation and aberrant osteogenic differentiation,^44^ and serum starvation was reported to regulate autophagy in human periodontal ligament cells via the ROS‑AMPK‑mTOR axis.^45^ Although these findings pertain to periodontal tissues, they echo the concept that localized stress cues such as hypoxia or mechanical loading orchestrate catabolic and inflammatory programs relevant to early TMJOA. Intriguingly, the stabilizing effect of DMOG on HIF-1α notably decreased the expression of inflammatory cytokines and catabolic enzymes, while the knockdown of HIF-1α aggravated these effects. These results highlight the dual characteristics of HIF-1α: although pathological hypoxia promotes inflammation and matrix degradation, HIF-1α itself acts as a protective transcription factor,^46^ maintaining the homeostasis of chondrocytes. This is in accordance with previous findings indicating that under hypoxic conditions, HIF-1α supports matrix synthesis and cell survival.^47^, ^48^, ^49^ Our in vivo experimental results further confirm this protective effect, as DMOG treatment ameliorated cartilage degeneration in TMJOA induced by UAC, reduced the OARSI score, and restored subchondral bone structure. However, this study presents a novel perspective: HIF-1α is not just a proinflammatory factor in the early stage of TMJOA; it also exerts via MDM2-mediated suppression of the p53 signalling pathway, which significantly differs from the conclusions of some studies on end-stage disease that suggest HIF-1α promotes degeneration.^50^ This disparity may originate from differences in the stages of the research models. This study focuses on the inflammatory response induced by a hypoxic environment, whereas previous studies predominantly observed the inflammatory response induced under normoxic conditions. Additionally, the establishment of the HIF-1α/MDM2/p53 axis association for the first time in this study supplements the molecular network details of hypoxia regulation in TMJOA and offers direct evidence supporting the viewpoint that hypoxia is both a driving factor and a potential therapeutic target.^51^^,^^52^

The scientific significance of this study lies in redefining the role of hypoxia in TMJOA: it is not merely a pathological by-product but also an active initiator of early inflammation and matrix degradation. The protective regulatory mechanism of HIF-1α (transactivating Mdm2 to elevate MDM2 protein and suppress p53 signalling) provides a novel target for intervention stabilizing HIF-1α can effectively alleviate cartilage degeneration, reduce OARSI scores, and repair subchondral bone structure. Clinically, these findings pave the way for a multipronged translational strategy. Firstly, diagnostically, the identification of the HIF-1α/MDM2/p53 axis offers specific biomarkers for early disease detection. Given the asymptomatic nature of early TMJOA, monitoring the levels of stabilized HIF-1α or specific p53 isoforms in synovial fluid or serum via minimally invasive arthrocentesis could serve as a liquid biopsy approach to identify high-risk patients before irreversible structural damage occurs.^53^ Secondly, therapeutically, while systemic administration of PHD inhibitors (like DMOG) carries risks of off-target effects (eg, polycythaemia), our data support the development of localized delivery systems. We envision that intra-articular injections of nano-encapsulated DMOG derivatives or biocompatible hydrogels loaded with HIF-1α stabilizers could sustainably release drugs within the joint space, maintaining effective local concentrations while minimizing systemic exposure.^54^ Furthermore, gene therapy approaches, such as adeno-associated virus-mediated *Mdm2* overexpression specifically in chondrocytes, represent a potential long-term solution to tip the balance from p53-driven catabolism back to homeostasis.

While the concordant results from siRNA-mediated HIF-1α knockdown strongly support a specific, protective role for HIF-1α in mitigating early hypoxic injury in TMJOA, it is important to acknowledge certain limitations. First, DMOG, as a broad‑spectrum PHD inhibitor, may also affect HIF‑2α and other 2‑OG‑dependent dioxygenases.^55^ Although we observed a consistent phenotype using siRNA, future studies employing more selective PHD inhibitors or conditional genetic models would help to further dissect the specific contribution of HIF‑1α stabilization. Second, this study focused on the early hypoxic phase of TMJOA. Whether HIF‑1α stabilization remains protective in later disease stages, where the hypoxic microenvironment may be altered by aberrant angiogenesis,^56^ requires further investigation. Lastly, while we confirmed HIF-1α exerts chondroprotection via the MDM2/p53 regulatory axis and identified the involvement of the p53 signalling pathway, the precise molecular mechanisms by which it mediates chondrocyte catabolism and the specific downstream effector molecules involved demand more in‑depth exploration.

Collectively, this study reveals that hypoxia in early TMJOA plays a dual role: while pathological hypoxia aggravates cartilage inflammation and matrix degradation, stabilizing HIF-1α under hypoxic conditions exerts chondroprotective effects via MDM2-mediated p53 ubiquitination and degradation, thereby mitigating inflammatory response and matrix catabolism. Clinically, this suggests early detection of hypoxia biomarkers and targeted HIF-1α modulation could delay TMJOA progression. Future research should validate these findings in advanced vascularized TMJOA models and elucidate the downstream effectors of the HIF-1α/MDM2/p53 axis to refine therapeutic strategies.

## Author contributions

*Contributed equally to conception, design, data acquisition, analysis, and interpretation, and drafted the manuscript:* Chen, Cai. *Contributed to data acquisition and analysis:* Cai, Yang. *Contributed to data acquisition and analysis:* Z. Wang, Chen. *Contributed to data analysis and interpretation:* Chen. *Contributed to conception and design, data interpretation, and critical revision of the manuscript:* Xu, H. Wang. *Final approval and agreed to be accountable for all aspects of the work:* All authors.

## Conflict of interest

The authors declare that they have no known competing financial interests or personal relationships that could have appeared to influence the work reported in this article.

## Appendix Supplementary materials

Image, application 1

## Footnotes

Supplementary material associated with this article can be found in the online version at doi:10.1016/j.identj.2026.109805.
