# Supplementary material for: Hypoxia-Inducible Factor-1α Stabilization Alleviates Hypoxia-Induced Temporomandibular Joint Osteoarthritis by Activating MDM2 to Suppression of p53 Signalling
Source: Int Dent J. 2026 Aug 22;76(5):109805. doi: 10.1016/j.identj.2026.109805 (PMC13524871; doi:10.1016/j.identj.2026.109805)
Supplement: Supplementary file 1 [file mmc1.docx]

**Fig. S1** Supplementary functional detection of chondrocytes under normoxia and hypoxia conditions. (**a**) Representative immunofluorescence images of (green, 488 nm) and (red, 647 nm) in condylar cartilage. NC: Negative control stained with PBS instead of primary antibodies. Scale bars = 50 μm. (**b**) CCK-8 assay for relative cell viability of chondrocytes treated with gradient concentrations of DMOG (0, 0.1, 0.5, 1, 2, 4 mM). (**c**) Annexin V/PI flow cytometric analysis of chondrocyte apoptosis after 24 h normoxic or hypoxic culture. (**d**) Quantification of relative pimonidazole fluorescence intensity reflecting intracellular hypoxic level in NX and HX groups. (**e**) CCK-8 assay for relative cell viability of chondrocytes cultured under normoxia or hypoxia for 24 h. (**f**) Toluidine blue staining detecting sulfated glycosaminoglycan deposition in chondrocytes cultured under normoxia or hypoxia for 7 days.Data are presented as mean ± SEM (n = 3 per group). Statistical significance was determined by unpaired Student’s t-test. ns, no significance. ****p* < 0.001.

**Fig. S2** HIF-1α stabilization suppresses inflammatory response and matrix degradation in condylar cartilage under normoxic conditions. (**a**-**b**) Western blot images and quantitative analysis of HIF-1α, iNOS, MMP13, and TNF-α protein levels in chondrocytes under normoxic and normoxic with DMOG (NX+DMOG) conditions. (**c)** Relative mRNA expression levels of *Il6*, *Nos2*, *Mmp13*, and *Tnf* in chondrocytes under NX and NX+DMOG conditions, normalized to the NX group. (**d**) Representative immunofluorescence images and quantitative analysis of IL-6 and MMP3 in chondrocytes under NX and NX+DMOG conditions. Scale bar: 50 μm. (**e**-**f**) Western blot images and quantitative analysis of HIF-1α, iNOS, MMP13, and TNF-α protein levels in chondrocytes under normoxic and normoxic with transfection of NC or two different HIF-1α-specific siRNAs (siHIF-1α 1, siHIF-1α 2). (**g**) Relative mRNA expression levels of *Il6*, *Nos2*, *Mmp13*, and *Tnf* in chondrocytes transfected with HIF-1α-specific siRNA or NC under hypoxia. (**h**) Representative immunofluorescence images and quantitative analysis of IL-6 and MMP3 in chondrocytes transfected with HIF-1α-specific siRNA or NC under hypoxia. Scale bar: 50 μm. Data are presented as mean ± SEM (n = 3 independent experiments). Statistical significance was determined by unpaired Student’s t-test for comparisons between two groups (panels **a**-**d**), or by one-way analysis of variance (ANOVA) followed by Tukey’s post hoc test for comparisons among three or more groups (panels **e**-**h**). **p* < 0.05, ***p* < 0.01, ****p* < 0.001, *****p* < 0.0001.

**Fig. S3** (**a**) TUNEL immunofluorescence staining detecting apoptotic chondrocytes in condylar cartilage of Sham, UAC and UAC+DMOG groups; DAPI stained cell nuclei, scale bar: 50 μm. (**b**) ChIP-qPCR analysis of HIF-1α enrichment on the promoter of canonical target gene *Vegfa* (positive control) in chondrocytes cultured under hypoxic conditions with or without DMOG intervention, presented as percentage of input DNA (n = 3).
